# The prevalence of novel psychoactive substances (NPS) use in non-clinical populations: a systematic review protocol

**DOI:** 10.1186/s13643-016-0375-5

**Published:** 2016-11-21

**Authors:** Salma M. Khaled, Elizabeth Hughes, Dan Bressington, Monica Zolezzi, Ahmed Radwan, Ashish Badnapurkar, Richard Gray

**Affiliations:** 1 Social and Economic Survey Research Institute, Qatar University, Doha, Qatar; 2 Applied Mental Health Research, The University of Huddersfield, Huddersfield, UK; 3 Hong Kong Polytechnic University, Kowloon, Hong Kong; 4 Clinical Pharmacy and Practice, College of Pharmacy, Qatar University, Doha, Qatar; 5 Psychiatry Residency Program, Hamad Medical Corporation, Doha, Qatar; 6 Health Services and Population Research Centre, Hamad Medical Corporation, Doha, Qatar

**Keywords:** Novel psychoactive substances, New psychoactive substances, Legal highs, Research chemicals, Designer drugs, Internet drugs, Club drugs, Epidemiology, Prevalence, Psychiatric morbidity

## Abstract

**Background:**

Novel psychoactive substances (NPS) are new narcotic or psychotropic drugs that are not controlled by the United Nations drug convention that may pose a serious public health threat due to their wide availability for purchase on the internet and in so called “head shops.” Yet, the extent of their global use remains largely unknown. The aim of this study is to conduct a systematic review of the prevalence of NPS use in non-clinical populations.

**Methods:**

This is a systematic review of observational studies. Embase, MEDLINE, PubMed, Cumulative Index to Nursing and Allied Health (CINAHL), Cochrane Library, Lilacs, Scopus, Global Health, PsychINFO, Web of Science, and the World Health Organization (WHO) regional databases will be searched for eligible prevalence studies published between 2010 and 2016. Data from cross-sectional studies that report the prevalence of NPS use (one or more types) in participants (of any age) from censuses or probabilistic or convenience samples will be included. Data will be extracted from eligible publications, using a data extraction tool developed for this study. Visual and statistical approaches will be adopted instead of traditional meta-analytic approaches.

**Discussion:**

This review will describe the distributions of various types of prevalence estimates of NPS use and explore the impact of different population groups and study-related and tempo-geographical variables on characteristics of these distributions over the period of 2010 to 2016.

**Systematic review registration:**

PROSPERO CRD42016037020

## Background

### Definitions, types, and effects

The authors of the United Nations Office on Drugs and Crime report define novel psychoactive substances (NPS) as “substances of abuse, either in a pure form or a preparation, that are not controlled by the 1961 Single Convention on Narcotic Drugs or the 1971 Convention on Psychotropic Substances, but which may pose a public health threat” [1]. “Spice,” “bath salts,” “Special K,” and “herbal incense” are a few street names for a whole new milieu of “designer” drugs that have seen a dramatic increase in use across the globe. Part of the problem in controlling the proliferation of these substances lies in their variety, their ease of synthesis, low cost, being undetectable by standard toxicology screens, and resourceful marketing. In addition to being available from drug traffickers, they are often sold on the internet as well as in neighborhood head shops [2].

Although the NPS terminology seems to imply the creation of novel drugs, many are not new. In fact, most psychoactive drugs (that later became abused) have been in use for at least a century. Many NPS were discovered at the same time as other drugs, and it was simply, for whatever reasons, these other drugs became popular. The majority of these substances are chemicals produced by tweaking or altering the molecular structure of previous well-known psychoactive agents such as cannabis, cocaine, methylenedioxymethamphetamine (or MDMA, commonly known as ecstasy), and lysergic acid diethylamide (or LSD), which are being sold as “legal highs,” “research chemicals,” “herbal highs,” “party pills,” or “plant food” in an attempt to stay ahead of the law prohibiting the sale and use of psychoactive drugs.

NPS are formulated in a variety of forms, and when sold in the market, many contain mixtures of different substances some including controlled drugs. Smoked, ingested, snorted, or injected NPS can produce a variety of psychotropic effects, which can be similar to those related to the controlled substances they contain or to the chemical entities that they are derived from. Based on their clinical effects, NPS can be classified as stimulants, empathogens-entactogens, sedative-hypnotic-anxiolytics, dissociatives, and hallucinogens. Alternatively, they can be classified according to their chemical class as phenethylamines, piperazines, tryptamines, synthetic cathinones, alkylindoles (synthetic cannabinoids), and arylcyclohexylamines [3].

Pharmacologically, these substances affect dopamine, noradrenaline, and serotonin producing a broad spectrum of effects [1, 4]. Additionally, their potential for addiction and toxic effects is diverse and varies depending on the type of NPS. Research on the health implications of most NPS is limited, but what evidence there is suggests that the adverse effects can be just as serious as for controlled drugs. In fact, a broad range of negative health outcomes have been associated with their use [1, 3, 5]. These can be physical or psychological, including cardiovascular problems, seizures, renal failure, myocardial infarction, anxiety, agitation, memory loss, depression, and psychosis [6–11]. Health impacts also depend upon whether other drugs and/or alcohol are used at the same time as NPS and the method of use (if they are swallowed, snorted, or injected). Deaths have been associated with drugs in each of the main NPS groups [12].

### Emergence of NPS

Since 2010, there has been a marked increase in the new types of NPS that have been detected for the first time with over 70% of these substances detected in the last 5 years alone [13]. From 166 new substances reported in 2009 to 348 in 2012 [14] and 450 in 2014 [15], in 2015, the European Monitoring Centre for Drugs and Drug Addiction detected over 100 new psychoactive substances bringing the total number of monitored NPS to more than 560 [16]. In fact, their widespread availability through the internet made the issue of NPS a global phenomenon with evidence that the majority of countries that reported the emergence of NPS were from Europe, followed by Asia, Africa, the Americas, and Oceania [12].

### Estimating prevalence of self-reported use through surveys

Despite great concern about the increasing availability worldwide and their potential adverse effects, there has been little consideration of the public health burden associated with NPS use. Prevalence is an epidemiological parameter critical for generation of burden metrics, such as the disability-adjusted life year (DALY), now largely used by governments for prioritization of health care and service planning [17].

To collect information about the prevalence of NPS use in the general population, several countries have recently opted for the inclusion of NPS in routine national drug surveys. Based on data from limited number of countries (Canada, USA, Australia, New Zealand, and Ireland), the estimated 12 months prevalence of NPS use for 2011–2012 period ranged from 0.4 to 5.9% [18–23]. In the US, more recent national use estimates are available from two main sources: Monitoring the Future (MTF) study and the National Survey on Drug Use and Health (NSDUH). MTF reported 12 months prevalence of synthetic cannabinoids and bath salts of 10 and 1%, respectively [24]. Meanwhile, data from five cohorts in the NSDUH sampled from households between 2009 and 2013 reported a lifetime prevalence of 1.2% of any NPS use. Psychedelic tryptamines (86.1%), psychedelic phenethylamines (3.9%), and synthetic cannabinoids (3.6%) were the top three most widely used NPS types over this time frame [25].

Although most of the epidemiological data on NPS use come from general population surveys, it is known that some groups of users, particularly some marginalized groups, may be underrepresented in these surveys. This constitutes a major limitation of these surveys given that NPS use is concentrated in certain subgroups such as the young [26], the mentally ill [27], and other special groups (clubbers, psychonauts (people who use substances for exploratory purposes), and people who are lesbian, gay, bisexual, and transgender (LGBT)).

Alternative approaches to NPS prevalence estimation in non-clinical samples (country-specific and international) utilize “flash surveys” often online-, but also phone-based surveys, which obtain results relatively quickly through purposive sampling with focus on specific target groups. Although largely limited by self-selection, surveys like the Flash Eurobarometers and Global Drugs Survey (GDS) have sample sizes of users that are considerably bigger than most national household surveys. Therefore, provided estimates from subgroups of users that may be largely underrepresented in more “traditional surveys”—those based on census or probability samples. For example, the GDS captures NPS use among thousands of clubbers, psychonauts, and students, thus providing valuable information related to use in at-high risk subpopulation groups. According to the most recent GDS, an 11.6% rise in last year purchase occurred in the USA, the highest rate of any country assessed. The next highest estimates were found in the US, Canada, and the Netherlands with the lowest estimate in Switzerland. Globally, the mean percentage of NPS use was 4.8% [28].

Beyond sample designs and characteristics of participants, there are many challenges related to the evolving nature of NPS including the lack of universal definition of what constitutes an NPS, the large and increasing number of substances regarded as NPS, and the differences in legislation between countries. These issues are too complex to be adequately controlled by any one survey or a handful of large-sized surveys, if at all.

### Review aims

The aim of this review is to identify and collate studies describing the prevalence of NPS use over the period of 2010 to 2016. To achieve this aim, we will undertake a systematic review of studies reporting point, period, and lifetime prevalence estimates of NPS use in non-clinical populations. We will evaluate the quality of studies and the reported data, which will be extracted and filtered sequentially using multiple criteria and decision rules defined a priori. Data will be synthesized and described using cumulative prevalence distribution plots and related summary measures. The role of temporal, geographical, and other study level variables as potential sources of heterogeneity will also be explored graphically and through planned statistical comparisons.

### Search strategy

There is currently no universal definition for novel psychoactive substances (NPS). Furthermore, the extent of variability in reported prevalence estimates of use is largely attributed to lack of such consensus. To balance this need for inclusiveness with practical constraints related to resources, we will use the definition proposed by the United Nations Office on Drugs and Crime [1] to define novel psychoactive substances (NPS). As our interest lies mostly in self-identified NPS use, prevalence as an epidemiological parameter, and population-based surveys as methodological framework for capturing this outcome of interest, we will use layman search terms suggested in a recent review [3].

Search terms will include the following:

(“new psychoactive substances” OR “novel psychoactive substances” OR “legal highs” OR “designer drugs” OR “research chemicals” OR “smart drugs” OR “emerging drugs of abuse” OR “club drugs” OR “herbal highs” OR “bath salts” OR “internet drugs” OR “spice” OR “synthetic drugs”) AND (“prevalence” OR “epidemiology “OR “rate”)

The search string was developed using the Embase (Ovid) database and lists a number of relevant medical subject headings (MESH) and free text words for each construct. The search terms within each concept will be combined using the Boolean operator “or” and the terms across the two key concepts will be combined with “and.” We will then adapt the search strings for other electronic databases as necessary.

In order to identify the relevant papers, we will search the following databases:

- EMBASE/OVID (2010–2016)
- MEDLINE/OVID (2010–2016)
- Cumulative Index to Nursing and Allied Health (CINAHL with Full Text) (2010–2016)
- The Cochrane Library (2010–2016)
- LILACS
- Scopus
- PubMed
- Global Health
- PsychINFO
- Web of Science
- World Health Organization (WHO) regional databases including Index Medicus for the Eastern Mediterranean Region (IMEMR), African Index Medicus (AIM), Index Medicus for South-East Asia Region (IMSEAR), and Western Pacific Region Index Medicus (WPRIM)

We will search all relevant journals and manually review the reference lists of included articles to identify additional studies. Proceedings from scientific meetings and conference abstracts will also be included. A “Google” search will be performed to identify relevant reports published by international non-government organizations (e.g., WHO, United Nations) and drug monitoring agencies as well as local government reports. In addition, we will initiate contact via email with scientific authorities in the field of substance abuse from North America, South America, Europe, Africa, Asia, Oceania, and the Middle East to ascertain their awareness of relevant unpublished reports or studies in progress. Only articles or scholarly works or reports available in English, German, French, Spanish, Arabic, and Portuguese will be included.

### Inclusion and exclusion criteria

We will include cross-sectional probability or census-based surveys that report on prevalence of any type of NPS use in community dwelling individuals. We will exclude studies based on inpatient clinical populations. Studies that do not report prevalence of NPS use or information that would allow estimation of any type of prevalence parameters will be excluded. If relevant data necessary for calculating the precision of the prevalence estimates are missing from the original publications, we will request this data directly from authors. Data from international drug monitoring agencies or online surveys will be included if available.

### Condition or domain being studied

Any type of NPS use will be considered with or without other substance use disorders. Self-identification will be the primary method of case ascertainment in the population of interest. Studies using recognized diagnostic criteria, standardized instruments, or biological tests for ascertainment of NPS use will also be included.

### Participants/population

The review is concerned with respondents of any age from non-referred samples: the general population or community or educational institutions (schools, colleges, and universities) with a self-reported history of recent NPS use including initiation and recurrent use.

### Intervention(s), exposure(s)

None.

### Comparator(s)/control

None.

### Context

Prevalence estimates will be computed based on a review of studies from North America, South America, Europe, Africa, Asia, Oceania, and the Middle East conducted from 2010 to 2016. Countries will be categorized as low, middle, and high income based on the World Atlas Method [29].

### Outcome(s)

The prevalence of NPS use is defined as the proportion of users of any type of NPS at a given point in time (point prevalence) or over a specified period of time (period prevalence) or the proportion of ever users (lifetime prevalence). Synthesis of data related to the prevalence of any type of NPS is the primary outcome for this review. Synthesis of data related to prevalence of commonly (across maximum number of studies) defined classes of NPS would be carried out on ad hoc basis. Synthesis of data related to the prevalence of specific NPS is out of the scope of the current review.

### Data extraction, sorting, and selection

Abstracts identified in the searches will be exported to reference management software. Duplicate records will be excluded (using endnote and manually). Titles and abstracts of all identified articles will be screened for eligibility based on the following criteria: (1) significant reviews of the literature reporting NPS use prevalence; (2) major reports on NPS; and (3) original investigations on prevalence of substance use in non-clinical samples of participants of any age. Two members of the research team will conduct the screening process independently. Disagreements will be resolved by discussion with the remaining members of the research team and/or an independent researcher as necessary. An eligibility form will be developed based on the inclusion/exclusion criteria for this review, and reasons and information about included/excluded studies will be recorded. A similar process will be followed for the full-text screening stage of the review.

We will develop a data extraction tool, which will be piloted on a random sample of papers to check for accuracy and consistency. The following information about each study will be extracted:

1. Study or report characteristics: author (s), study year, country, publication type, language(s)
2. Study design/methodology: study design, sample size, sampling strategy, recruitment methods, sample coverage (community, country, region), survey administration modality
3. Parameter of interest: prevalence type including point-, period-, lifetime-prevalence, and non-specified. Type of parameter information available (numerator and denominator or prevalence and denominator or prevalence and standard error, or prevalence and 95% confidence intervals)
4. NPS-related information: definition(s), wording of use questions, level of information discrete drugs, drug classes, or no information, sources of reported NPS use (proxy or non-proxy), point of purchase
5. Special groups: information on type of population groups (students, psychiatric morbidity clubbers/ravers, psychonaut, and LGBT
6. Country-level variables: country’s economic status/measure of wealth, geographical region
7. Person-level variables: age and gender

Included studies will be categorized and sequentially filtered by a series of criteria including population group (households, schools, and other special groups), survey mode (face-to-face, phone, internet), sample type (probability, census, convenience), prevalence type (point, period, lifetime, and non-specified), and NPS type (any NPS versus specific NPS types—chemical drug name, chemical class name, “street” name). Rules will be developed, pretested, and systematically applied to allow careful selection of prevalence data without counting the same individuals more than once within the same study or in different studies. Specifically, selection rules that prioritize the inclusion of the single most informative and/or representative prevalence estimate will be developed and applied to situations where overall (combined) and class-specific prevalence estimates of NPS are reported by the same study. These rules will be developed ad hoc (if we find that most studies were reporting the prevalence of specific classes—e.g., synthetic cannabinoids, synthetic cathinones—as opposed to any NPS use then when we come across a study that reports both, we will choose the former type of estimates).

Prevalence estimates will be extracted separately for those with and without psychiatric morbidity. To avoid duplication, where person- and psychiatric-specific estimates are reported by the same study, only the latter will be included in the analyses.

Age-specific or overall age prevalence estimates (range) will be extracted separately. To ensure independence of observations, where both are reported, only the latter will be included. Where only age-specific estimates are reported, these will be combined to calculate the overall age prevalence by summing the number of cases across each age group and dividing by the summed denominator across each age group.

### Risk of bias (quality) assessment

A tool specifically designed for the assessment of the risk of bias and methodological quality of population-based prevalence studies will be used for the purpose of this review [30]. Studies will be graded using this tool, which will be adapted to incorporate established criteria for high-quality surveys as per evaluation framework put forth by the American Association of Public Opinion Research [31]. Studies will also be graded for the quality of reporting including information about how NPS was defined and operationalized.

### Strategy for data synthesis

Setting aside variability due to methodological issues related to quality of studies, reporting, and measurement, we anticipate that the underlying frequency distributions of NPS used to be largely heterogeneous. Prevalence estimates of NPS use are expected to vary widely between countries/regions and time periods due to differences in population characteristics and socio-economic and exposure levels. Therefore, traditional meta-analytic approaches of pooling data to produce a single estimate of prevalence may be conceptually problematic, potentially contributing to loss of informative variation about the prevalence of NPS use. For this reason, we will use descriptive approaches to reporting variations in the distributions of different types of prevalence data across studies. Cumulative prevalence distributions will be plotted for each prevalence type (point, period, and lifetime) and summary statistics for each plot will be provided in accompanying tables with quantiles (including median, mean, interquartile range, and standard deviations). Potential sources of heterogeneity can be investigated through sorting the data according to various rules and comparing the resulting distributions i.e., assessment of significant distributional changes in the cumulative prevalence plots resulting from the inclusion/exclusion of different subsets of studies [32].

In this respect, we plan to conduct a series of sensitivity analyses related to understanding variation in NPS use prevalence by country-level variables (economic status, region), population groups (students, people with psychiatric comorbidity, ravers/clubbers, and people who are gay or lesbian), and study-level variables (survey mode, sample type). Additionally, we aim to understand the role of changing definitions of NPS by detecting significant differences in the distribution of prevalence estimates when data are sorted by studies applying different definitions of NPS. Comparisons between the distributions of proportion estimates for different groups (e.g., males versus females) or for different levels of other variables of interest will be described using the ratio of the measures of central tendency for each variable level (e.g., male to female prevalence estimate ratio). Necessary multiple-comparison adjustments to the tests of significance will be carried out for any post hoc analyses.

Temporal trends will also be investigated by visually inspecting and testing for significant distributional changes in the cumulative prevalence plots and corresponding changes in the effect size of the prevalence estimates in response to the stepwise addition of studies after sorting data in chronological order [33]. This allows estimation of the contribution of studies over time.

## Discussion

This systematic review will produce and describe prevalence distributions of NPS use based on data collected by studies conducted between 2010 to 2016 in North America, South America, Europe, Africa, Asia, Oceania, and the Middle East. This review will also explore study-, person-, group-, and NPS-related variables contributing to the heterogeneity of these distribtutions providing a much-needed picture of the extent of variation in prevalence of NPS use across different populations and regions of the world since 2010. Researchers, policy makers, and public health stakeholders will use evidence resulting from the study to set research, policy, and program priorities for surveillance purposes, prevention, and provision of adequate clinical services. This information will also provide necessary insight into the effects of international efforts at policy and control of NPS by identifying “hot spots” and potential “at risk populations,” detailing the relative role of environmental factors in the formation of problematic NPS use.

## Abbreviations

AIM: African Index Medicus
CI: Confidence intervals
IMEMR: Index Medicus for the Eastern Mediterranean Region
IMSEAR: Index Medicus for South-East Asia Region
MESH: Medical subject headings
NPS: Novel psychoactive substances
PRISMA: Preferred Reporting Items for Systematic Reviews and Meta-Analyses
SE: Standard error
WHO: World Health Organization
WPRIM: Western Pacific Region Index Medicus
